# Performance of Diesel Engine Using Diesel B3 Mixed with Crude Palm Oil

**DOI:** 10.1155/2014/531868

**Published:** 2014-02-05

**Authors:** Nattapong Namliwan, Tanakorn Wongwuttanasatian

**Affiliations:** ^1^Department of Mechanical Engineering, Faculty of Engineering, Khon Kaen University, Khon Kaen 40002, Thailand; ^2^Centers for Alternative Energy Research and Development (AERD), Khon Kaen University, Khon Kaen 40002, Thailand

## Abstract

The objective of this study was to test the performance of diesel engine using diesel B3 mixed with crude palm oil in ratios of 95 : 5, 90 : 10, and 85 : 15, respectively, and to compare the results with diesel B3. According to the tests, they showed that the physical properties of the mixed fuel in the ratio of 95 : 5 were closest to those of diesel B3. The performance of the diesel engine that used mixed fuels had 5–17% lower torque and power than that of diesel B3. The specific fuel consumption of mixed fuels was 7–33% higher than using diesel B3. The components of gas emissions by using mixed fuel had 1.6–52% fewer amount of carbon monoxide (CO), carbon dioxide (CO_2_), sulfur dioxide (SO_2_), and oxygen (O_2_) than those of diesel B3. On the other hand, nitric oxide (NO) and nitrogen oxides (NO_*X*_) emissions when using mixed fuels were 10–39% higher than diesel B3. By comparing the physical properties, the performance of the engine, and the amount of gas emissions of mixed fuel, we found out that the 95 : 5 ratio by volume was a suitable ratio for agricultural diesel engine (low-speed diesel engine).

## 1. Introduction

Nowadays, the energy consumption of Thailand has been rising according to the growth of population, economy, and industrial sectors [1]. The report on the energy consumption states that, in the year 2011, Thailand energy consumption has increased by 7.3%. At the same time, the fuel demand increased by 8.7%; particularly the demand for the diesel fuel in the year 2010 has increased by 3.9%. Due to the high demand of diesel and the rapid rise in oil price, the Thai government has launched an alternative energy (based on local raw materials) development policy in order to enhance Thailand's energy security.

Thailand is an agricultural country; hence there are abundant choices for alternative energy materials, particularly vegetable oil and animal fat for biodiesel supply. Oil crops in Thailand are oil palm, coconut, soya bean, Jatropha, Castor bean, and so forth. Jenvanitpanjakkul [2] directly used crude and refined groundnut oil on 7 hp diesel engine with no engine modification. Both crude and refined groundnut oil resulted in start-up problems, rough running at low speed, incomplete combustion, and disruption of the engine. During short-term performance tests, the output power using crude and refined groundnut oil gave similar results compared to diesel fuel.

Sangsawang et al. [3] studied performance, exhaust gas emission and heat emission, tests on IDI (indirect injection) type diesel engine with 10% volume of crude palm oil blended with diesel. The results showed lower engine performance and higher toxic-gas emissions when using the blended fuel. Karaosmanoglu et al. [4] reported the performance of diesel engine run with sunflower oil at various speeds (rpm). Power, torque, average pressure, and black smoke emission were reduced while brake specific fuel consumption (bsfc) increased as compared to engine using pure diesel. Hitam and Jahis [5] reported the use of palm oil on Elsbett diesel engine running for 80,000 km. Average urban fuel consumption was 8 liters per 100 km and 7 liters per 100 km for extraurban. No troubles were found during the test with smooth-running and continuous fuel feed. No side effects were found even with parts of engine. Bari et al. [6] studied the effect of preheating crude palm oil on the injector system in order to reduce its viscosity. This research concluded that preheated palm oil at 60°C can suppress clogged fuel. The combustion analysis showed that the preheated oil can increase 6% maximum pressure as well as 2.6% combustion delay. Dorado et al. [7] studied the comparison between the toxic-gas emissions by waste olive oil biodiesel versus diesel in direct injection diesel engine. The results showed that the biodiesel gave lower amount of emissions in case of CO, CO_2_, NO, and SO_2_ while slightly higher in case of NO_2_ and had higher brake specific fuel consumption (bsfc) as compared to pure diesel. The combustion efficiency of both was almost the same. May et al. [8] used ethyl esters of crude palm oil as fuel to test the performance of 36 diesel engines running for the total distance of 300,000 km. No technical problems were found whether in performance, fuel consumption, for ash accumulation on parts. The results also showed that the emission of HC, NO_*X*_, and CO was lower and also none of SO_2_ was found. However, some parts of the engine which were made from low grade plastic or rubber were affected. Agarwal and Rajamanoharan [9] studied the performance and emission characteristics of a compression ignition engine fuelled with Karanja oil and its blends (10%, 20%, 50%, and 75%) with diesel and the effect of preheated oil on engine performances. The brake specific fuel consumption and brake specific energy consumption of the engine with preheated blends showed an improved trend. The unheated oil blends up to K50 also showed an improved trend compared to diesel. The smoke density from exhaust gas of preheated lower blends as well as unheated lower blends was almost similar to that of diesel fuel. The HC emissions from unheated and preheated lower blends (K10 and K20) are lower than that of diesel. The emission of NO from all blends with and without preheating is lower than diesel at all load conditions. This is a significant advantage over diesel while using vegetable oil and their blends. Hence, it can be concluded that the Karanja oil blends with diesel up to 50% (v/v) without preheating as well as with preheating would replace diesel for running the CI engine with lower emissions and improved performance. Canakci et al. [10] studied the comparison of preheated crude sunflower oil (PCSO) and petroleum based diesel fuel (PBDF) for tested combustion and emission properties by indirect injection (IDI) engine. The results showed that the cylinder gas pressure and heat release curves for PCSO at 75°C were similar to those of PBDF. The ignition delays for the PCSO were longer and the start of injection timing was earlier than for PBDF. The difference in the average brake torque was a decrease of 1.36% for PCSO. The brake specific fuel consumption increased by almost 5% more or less in proportion to the difference in calorific value. The emission test results showed that the decreases in CO_2_ emissions and smoke opacity were 2.05% and 4.66%, respectively. Leevijit and Prateepchaikul [11] studied the performance and emissions of an indirect injection (IDI)-turbo automobile diesel engine operated with diesel and blends of degummed-deacidified mixed crude palm oil. This work aimed to examine and compare the performance at various loads and speeds by using the difference ratio of blended fuel. The results showed that all blends produced the same maximum brake torque and power. A higher blending portion resulted in a little higher brake specific fuel consumption, a slightly lower brake thermal efficiency, a slightly lower exhaust gas temperature, and a significantly lower amount of black smoke. The level of carbon monoxide from the 20 vol.% blend was significantly lower and the levels of nitrogen oxides from all blends were a little higher.

Based on reviews, many researches focused on transesterified palm oil or preheated palm oil. This work aimed to use of crude palm oil blends without transesterification and preheating processes since the converting of crude oil to biodiesel process is costly and the obtained biodiesel properties are inconstant depending on the conversion process applied. However, using crude palm oil directly mixed with diesel is needed to investigate the limit of quantity of crude oil to be mixed in the blend affecting the engine performance and exhaust emission. The properties of blends, diesel engine performance, fuel consumption, and toxic-gas emission were studied. Diesel B3 was mixed with crude palm oil in the volume ratio of 95 : 5 (DP5), 90 : 10 (DP10), and 85 : 15 (DP15). The results of this study will be further used for alternative energy in diesel engine based on local raw materials development.

## 2. Research Methodology and Experimental Setup

### 2.1. Research Methodology

The research was carried out by mixing petroleum diesel B3 with crude palm oil in the volume ratios of 95 : 5, 90 : 10, and 85 : 15, respectively (Table 1). Diesel B3 is a commercial diesel fuel in Thailand which is a blend of pure diesel with 3% of ester-based biodiesel. The diesel engine performance, rate of fuel consumption, and toxic-gas emissions were investigated in order to determine the suitable volume ratio for agricultural machinery using diesel B3 as a reference.

### 2.2. Experimental Setup

**Figure 1:**
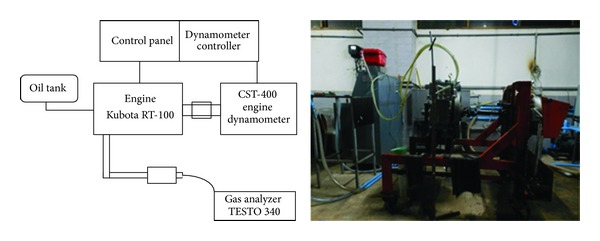
Diesel engine with hydraulic dynamometer performance testing.

The engine performance was tested on a direct injection single cylinder 4 stroke Kubota RT-100 diesel engine (volume = 547 cc) having maximum power of 7.5 kW, maximum torque of 3.6 kg-m @ 1600 rpm, and a fuel consumption rate of 240 g-hp/hr. The engine was connected with the hydraulic dynamometer CST-400 (Figure 1). Toxic-gas emissions (CO, CO_2_, NO, NO_*X*_, and O_2_) were analyzed using a gas industrial combustion analyzer-TESTO 340.

### 2.3. Method: Diesel Engine Performance Testing

The experiments started with engine performance testing by using diesel B3 as a reference and then using fuel blends in various ratios. The steps involved in the experiment are as follows.

1. Warm-up the engine for 20 minutes to prepare the engine, cooling systems, and lubrication systems before testing.
2. Set the maximum rotational speed (RPM) of the engine and then vary load of dynamometer until the speed descends to 2,400, 2,000, 1,600, 1,200, and 800 RPM. This strategy was to maintain air fuel ratio at each engine speed for each blend. Since the engine was not modified, the injection time for all tests was therefore fixed but the delay time varied as it depended on the type of fuel being used.
3. Monitor torque, fuel consumption (g), and toxic gas at each speed.
4. Use the other blend fuel ratio for testing. Notably, the remaining fuel had to be completely drained and emptied first from the engine before the new fuel blend was filled in. The filter must be replaced before testing another ratio of biodiesel blend.
5. Each experiment was repeated 3 times and the average values were used.

## 3. Results

### 3.1. Properties of Fuel Blends and Diesel B3

Some important properties of fuels were determined with marked standards. The properties are shown in Table 2. We found that blend with 95 : 5 ratio by volume shows the nearest properties to diesel B3.

### 3.2. Engine Performance Tests

#### 3.2.1. Torque and Power

**Figure 2:**
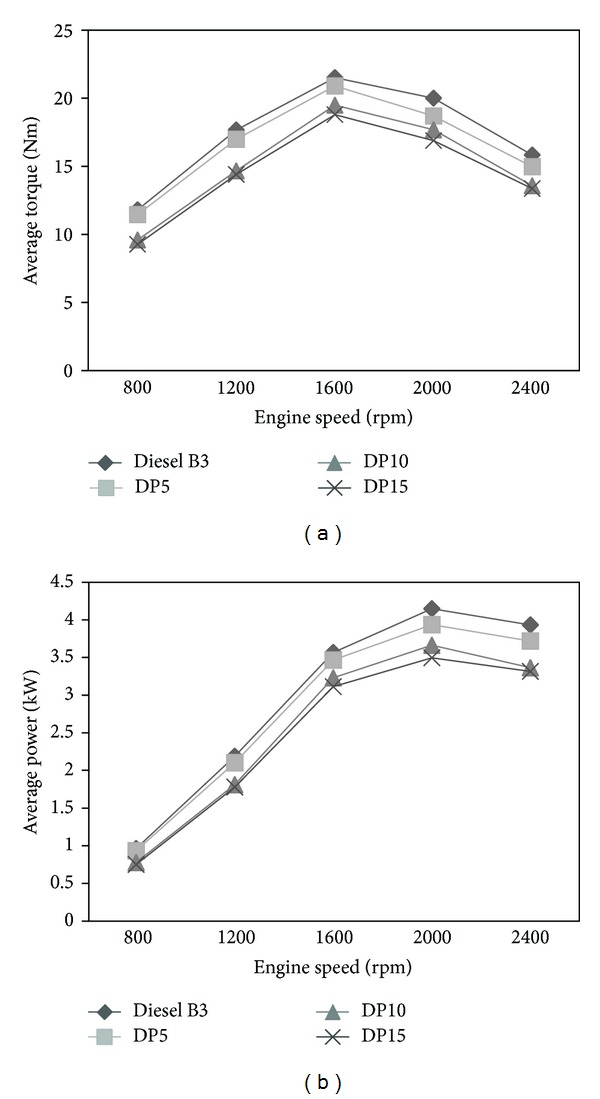
(a) Average torque versus RPM and (b) average power versus RPM.

Graphs of the engine torque versus engine speed of various types of fuels were plotted in Figure 2(a). The average torque of engine run with fuel blends DP5 (95 : 5), DP10 (90 : 10), and DP15 (85 : 15) was lower than that of diesel B3 by 5.6%, 14%, and 17%, respectively. The average power of engine run with mixed fuels was lower than the reference by 5%, 13%, and 15.7%, respectively. The reduction of torque and power of the engine, run with blends, was caused by the higher viscosity of the mixed fuel; hence there was difficulty in injection. This led to the delay time of combustion for blends resulting in drops in power. The conclusion of this work matches the research of Sangsawang et al. [3].

#### 3.2.2. Specific Fuel Consumption: SFC

**Figure 3:**
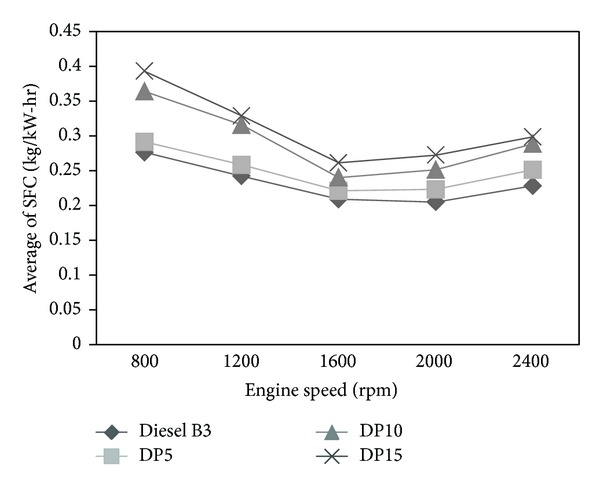
Average specific fuel consumption versus speed.

The SFC versus speed of various types of fuels was plotted in Figure 3. The average SFC of engine run with blends DP5, DP10, and DP15 was higher than that of diesel B3 by 7%, 25%, and 33%, respectively. Notably, SFC of mixed fuel increased with the increase in percentage of crude palm oil which has higher viscosity than diesel B3. Difficulty in injection caused by viscosity led to the incomplete combustion of fuel. The conclusion of this work also agrees with the report of Sangsawang et al. [3].

### 3.3. Toxic-Gas Emissions

#### 3.3.1. Carbon Monoxide (CO) and Carbon Dioxide (CO_2_) Emissions

**Figure 4:**
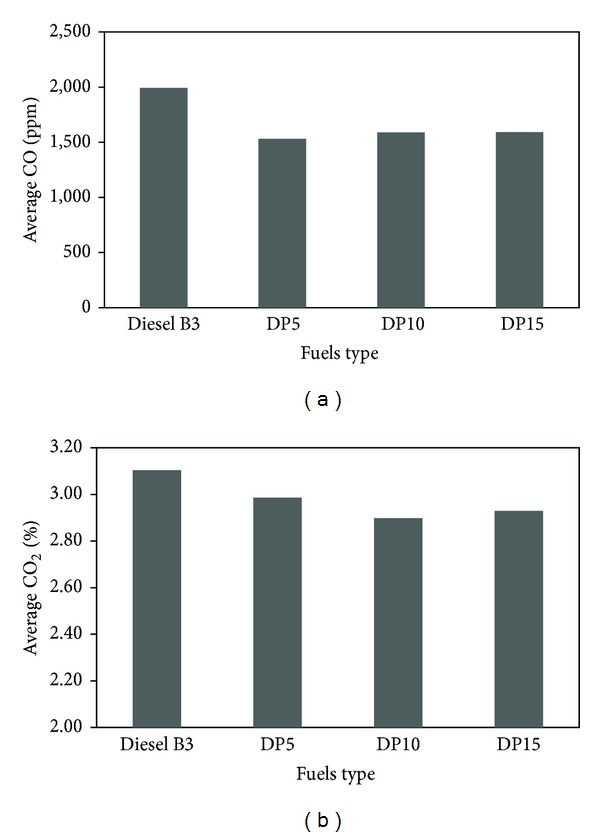
Average quantity of (a) carbon monoxide (CO) and (b) carbon dioxide (CO_2_).

Carbon monoxide (CO) and carbon dioxide (CO_2_) emissions of diesel engine run with various fuels were plotted in Figure 4. The average CO emission of DP5, DP10, and DP15 blends was less than that of diesel B3 by 23%, 20%, and 20%, respectively. The average CO_2_ emission of the blends was also less than B3 by 3.8%, 6.7%, and 5.6%, respectively. This can be explained since the fuel blends have less carbon composition than diesel B3; therefore, blends produce less toxic carbon monoxide and carbon dioxide. These results agree with Dorado et al. [7] and Raju et al. [12].

#### 3.3.2. Oxygen (O_2_) Quantity

**Figure 5:**
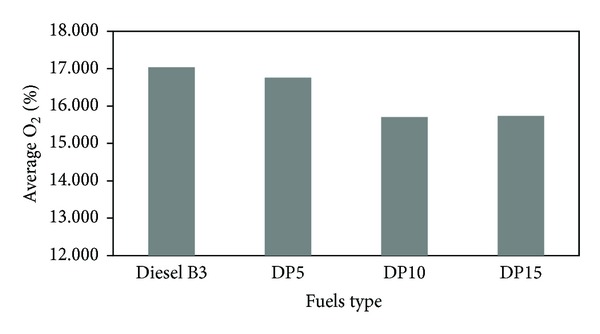
Average quantity of oxygen (O_2_).

Figure 5 demonstrates the oxygen (O_2_) emissions of diesel engine run with various types of fuel. We found out that the average O_2_ emissions of DP5, DP10, and DP15 mixed fuels were less than that of diesel B3 by 1.6%, 7.8%, and 7.6%, respectively. This is because the blends have more oxygen than diesel B3; hence, a more complete combustion can be achieved. Raju et al. [12] and Yusaf et al. [13] also reported the same phenomena.

#### 3.3.3. Nitric Oxide (NO) and Nitrogen Oxides (NO_*X*_) Emissions

**Figure 6:**
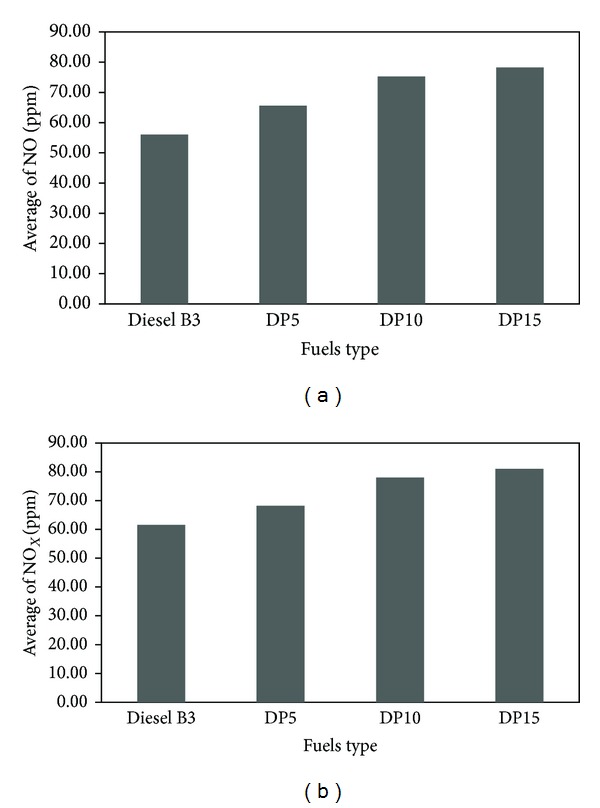
Average quantity of (a) nitric oxide (NO) and (b) nitrogen oxides (NO_*X*_).

Nitric oxide (NO) and nitrogen oxides (NO_*X*_) emissions of diesel engine run with various fuel types are illustrated in Figure 6. The average NO emission of DP5, DP10, and DP15 blends was found to be higher than that of diesel B3 by 17%, 34%, and 39%, respectively. For NO_*X*_, emission of blends was also higher than B3 with the average of 10%, 26%, and 31%, respectively. Due to higher temperature combustion of the mixed fuels than diesel B3, blends produced more nitric oxide and nitrogen oxides. This phenomenon was reported by many researchers, for example, Dorado et al. [7] and Prasad et al. [14].

#### 3.3.4. Sulfur Dioxide (SO_2_) Quantity

**Figure 7:**
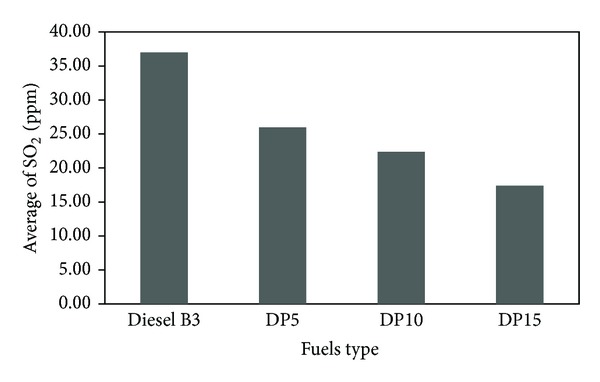
Average quantity of sulfur dioxide (SO_2_).

Sulfur dioxide (SO_2_) emission of diesel engine run with various fuel types is plotted in Figure 7. We found out that the average SO_2_ emission of DP5, DP10, and DP15 blends was less than that of diesel B3 by 30%, 40%, and 52%, respectively. The explanation for this is that palm oil has no sulfur content; hence mixed fuel with palm oil results in lower sulfur content in the blends. This leads to the reduction of sulfur dioxide emission. Similar results were found by Dorado et al. [7].

## 4. Conclusions 

This work studied the fuel properties, diesel engine performance, fuel consumption, and toxic pollutants of mixed diesel B3 with crude palm oil in the volume ratio of 95 : 5 (DP5), 90 : 10 (DP10), and 85 : 15 (DP15), respectively. The suitable ratio for agricultural (low speed) diesel engines was determined by using diesel B3 as a reference. The results of fuel properties, engine performance, fuel consumption, and toxic emission analysis concluded that the suitable volume ratio for agricultural diesel engines was 95 : 5 (DP5) due to the fact that its properties, performance, and specific fuel consumption were as good as diesel B3. Moreover, carbon monoxide (CO) and carbon dioxide (CO_2_) oxygen (O_2_) and sulfur dioxide (SO_2_) emission of DP5 blend was less than those of diesel B3.

Nevertheless, repeated experiments are required in order to solidify the results and conclusions. Moreover, further tests on performance, toxic emission, and other side effects on long-term use of the blends are being carried out, and also the deterioration of engine and fuel injection system and so forth are being investigated.

## Figures and Tables

**Table 1:** Volume ratio of petroleum diesel B3 : crude palm oil.

Biodiesel blend	Volume ratio (petroleum diesel B3 : crude palm oil)	Type
1	100 : 0	Diesel B3
2	95 : 5	DP5
3	90 : 10	DP10
4	85 : 15	DP15

**Table 2:** Heating values of glycerin and biomass raw materials.

Added	Properties	Standards	Types				Units
			Diesel B3	DP5	DP10	DP15	
1	Heat release rate	ASTM D240	45,805	45,429	44,988	44,761	kJ/kg
2	Viscosity at 40°C	ASTM D445	3.274	3.43	3.885	4.406	Centistokes (cSt)
3	Density at 15°C	ASTM D4052	841.9	844.6	848.0	851.4	kg/m^3^
4	Acid value	ASTM D664	0.105	1.455	2.825	3.600	Mg KOH/g
5	Cloud point	ASTM D2500	6.9	6.5	6.4	6.4	°C
6	Flash point	ASTM D93	67	66	70	69	°C
7	Gravity pour point	ASTM D79	−6.0	−15.0	−14.0	−8.0	°C
8	Water content	EN ISO 12937	69.5	130.65	182.15	233.95	mg/kg
9	Copper corrosion, 3 h at 50°C	ASTM D130	1A	1A	1A	1A	

Note: 1A is slight tarnish from copper corrosion.
